# Au/Fe_3_O_4_ core–shell nanoparticles are an efficient immunochromatography test strip performance enhancer—a comparative study with Au and Fe_3_O_4_ nanoparticles

**DOI:** 10.1039/c8ra00185e

**Published:** 2018-04-16

**Authors:** Jiaoling Huang, Zhixun Xie, Liji Xie, Zhiqin Xie, Sisi Luo, Xianwen Deng, Li Huang, Tingting Zeng, Yanfang Zhang, Sheng Wang, Minxiu Zhang

**Affiliations:** Guangxi Key Laboratory of Veterinary Biotechnology, Guangxi Veterinary Research Institute 51 You Ai North Road Nanning 530001 Guangxi China xiezhixun@126.com

## Abstract

Immunochromatography test strips that use metal particles constructed from Au, Fe_3_O_4_, and Au/Fe_3_O_4_ nanoparticles were developed for the rapid detection of avian influenza virus subtype H7 (AIV H7). The principle of this immunochromatography test strip was based on a sandwich immunoreaction in which AIV H7 antigens bind specifically to their corresponding antibodies on a nitrocellulose membrane. An antibody–metal (Au, Fe_3_O_4_ or Au/Fe_3_O_4_) nanoparticle conjugate was used as a label and coated onto a glass fiber membrane, which was used as a conjugate pad. To create a test and a control zone, an anti-H7 polyclonal antibody and an anti-IgG antibody were immobilized onto the nitrocellulose membrane, respectively. Positive samples displayed brown/red lines in the test and control zones of the nitrocellulose membrane, whereas negative samples resulted in a brown/red line only in the control zone. The limit of detection (LOD) of the Au/Fe_3_O_4_ nanoparticle-based immunochromatography test strips was found to be 10^3.5^ EID_50_ (EID_50_: 50% Egg Infective Dose), which could be visually detected by the naked eye within 15 min. In addition, 200 clinical samples were tested using the Au/Fe_3_O_4_ nanoparticle-based immunochromatography test strip to estimate its performance, and seven were positive for AIV H7. In summary, the Au/Fe_3_O_4_ nanoparticle-based immunochromatography test strip offers a simple and cost-effective tool for the rapid detection of AIV H7.

## Abstract 2

Immunochromatography test strips that use metal particles constructed from Au, Fe_3_O_4_, and Au/Fe_3_O_4_ nanoparticles were developed for the rapid detection of avian influenza virus subtype H7 (AIV H7).

## Graphical abstract

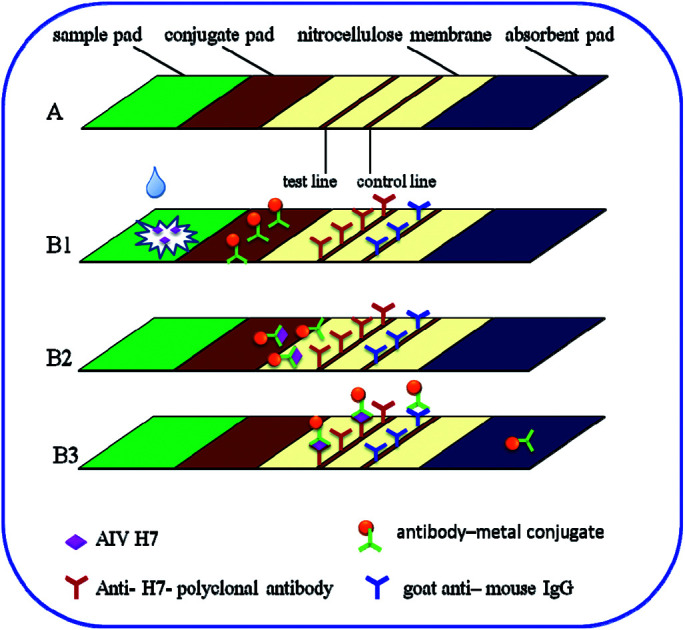

## Introduction

Avian influenza virus subtype H7 (AIV H7) has been frequently observed; for example, there was an AIV H7N2 outbreak in the Northeastern United States in 2002,^1^ and in the Netherlands, an AIV H7N7 outbreak not only impacted the poultry industry but also infected 89 people in 2003.^2^ In March 2013, the first case of human infection with avian influenza A H7N9 virus was reported by the Chinese Centers for Disease Control and Prevention, and since then, more than 450 human cases of H7N9 infection have been reported.^3,4^ A variety of technologies for diagnosing AIV H7, such as virus isolation and identification,^5^ reverse transcription-polymerase chain reaction (RT-PCR)-based assays,^6^ real-time reverse transcription-polymerase chain reaction (real-time RT-PCR),^7^ enzyme-linked immunosorbent assays (ELISAs)^8^ and reverse transcription loop-mediated isothermal amplification (RT-LAMP),^9^ have been developed. However, the disadvantages of these diagnostic methods, including the fact that they are time-consuming and require several experimental steps, including incubation and washing steps, make them less than ideal for practical applications. Therefore, it is necessary to explore simple, sensitive and rapid methods for the detection of virus AIV H7.

In the early 1980s, researchers developed immunochromatography test strips, which combine the advantages of chromatography and immunoassays into a single method. In this technique, the reaction between antibody and antigen occurs after chromatographic separation through a nitrocellulose membrane using capillary flow. Immunochromatography test strips are rapid, intuitive, user-friendly, inexpensive, and easily used by non-skilled personnel.^10–12^ Au nanoparticles have been the most widely used labels in immunochromatography test strips^12–17^ due to their long-term stability, easily controllable size distribution, and good compatibility with biological molecules, such as antibodies, antigens, proteins, DNAs, and RNAs. However, this method is generally only used for analyzing high concentrations of analytes. These limitations of Au nanoparticles have resulted in an increased use of various reporters that employ other nanoparticles as labels, such as magnetic nanoparticles (Fe_3_O_4_),^18^ organic fluorophores^19^ and quantum dots.^20^ Several studies have demonstrated that Fe_3_O_4_ particle-labeled detection systems specifically improve lateral flow assay sensitivity.^18,21^ Moreover, Fe_3_O_4_ particles are easily and rapidly separated using a magnet during the labeling process. However, the Fe_3_O_4_ particle surface must be modified prior to labeling.

Therefore, we hypothesized that Au/Fe_3_O_4_ core–shell nanoparticles could combine the advantages of Au nanoparticles and Fe_3_O_4_ nanoparticles and avoid the above-mentioned disadvantages of each of these particles. In the current study, we used Au/Fe_3_O_4_ core–shell nanoparticles as a label to develop a novel immunochromatography test for the detection of AIV subtype H7 (AIV H7) and compared these results to those obtained with Au and Fe_3_O_4_ nanoparticles.

## Materials and methods

### Ethics statement

This study was approved by the Institutional Animal Care and Use Committee (IACUC) of Guangxi Veterinary Research Institution. Sample collections were conducted based on the protocol #2017C110 issued by IACUC of Guangxi Veterinary Research Institution. Oral and cloacal swab samples were gently collected from poultry at various live bird markets. The fowls were not anesthetized before sampling and were observed for 30 min after sampling before being returned to their cages.

### Preparation of Au, Fe_3_O_4_ and Au/Fe_3_O_4_ core–shell nanoparticles

Fe_3_O_4_ and Au/Fe_3_O_4_ nanoparticles were prepared as previously described.^22^ Briefly, 4.64 g of FeCl_3_·6H_2_O and 1.71 g of FeSO_4_·7H_2_O (Guoyao Group Chemical Reagents Co., Ltd., Shanghai, China) were dissolved in 250 mL of deionized water at a Fe^2+^/Fe^3+^ molar ratio of 1 : 2, and 2 mL of 0.2 mol L^−1^ sulfuric acid was added to prevent the solution from undergoing Fe^2+^ oxidation. Ammonia (Guoyao Group Chemical Reagents Co., Ltd.) (25%) was added until the pH reached 9.0–9.5, and the solution was stirred for 30 min at room temperature. After amination, the mixture was heated to 80 °C and incubated for 30 min. A black suspension was produced, and this mixture was sonicated for 10 min. The deposit was separated using a magnet and rinsed with hot water until the fluid became neutral in color, yielding Fe_3_O_4_ nanoparticles. The Fe_3_O_4_ nanoparticles were then placed in a flask for preparation of the Au/Fe_3_O_4_ nanoparticles.

A total of 0.229 g of sodium citrate (Guoyao Group Chemical Reagents Co., Ltd.) was dissolved in 100 mL of deionized water, and the solution was heated to 99 °C using a water bath under vigorous stirring. A 1 mL volume of the prepared Fe_3_O_4_ suspension was then added to this solution. Finally, 5 mL of 10 mmol L^−1^ hydrochloroauric acid (Guoyao Group Chemical Reagents Co., Ltd.) was added dropwise to the solution, and the reaction was allowed to occur for 15 min. The water bath was then removed, and the suspension was continually stirred for 15 min. The solids were removed again using a magnet and rinsed with deionized water as the claret-red suspension was cooled to ambient temperature. An Au/Fe_3_O_4_ suspension was then prepared by dispersing the solids in 20 mL of water, which was maintained at 4 °C. Additionally, Au nanoparticles were obtained *via* the reduction of hydrochloroauric acid with sodium citrate using the same procedure but without addition of the Fe_3_O_4_ suspension.

### Preparation of the antibody–metal nanoparticle conjugates

The antibody–metal nanoparticle conjugates (antibody–Au, antibody–Fe_3_O_4_ and antibody–Au/Fe_3_O_4_ nanoparticle conjugates) were prepared according to a previously reported method^23,24^ with slight modifications. The Au/Fe_3_O_4_ nanoparticle solution was adjusted to pH 8.5 with 0.1 mol L^−1^ K_2_CO_3_, and 50 μL of anti-H7 monoclonal antibodies (Abcam, Cambridge, UK) (1 mg mL^−1^) was added dropwise to 10 mL of the Au/Fe_3_O_4_ nanoparticle solution. The mixture was incubated for 30 min at room temperature, and 1 mL of 10% bovine serum albumin (BSA) (Beijing Dingguo Biotechnology Co., Ltd., Beijing China) solution was then added to block the residual surface of the Au nanoparticles. The obtained solution was separated using a magnet, and after the supernatant was discarded, 1 mL of 1% BSA solution was added to the Au/Fe_3_O_4_ conjugate for resuspension. The separation and suspension processes were repeated twice, and the precipitate was resuspended in 2 mL of storage buffer (the storage buffer consisted of 50 mmol L^−1^ sodium phosphate buffer (pH 7.8) containing 5% polyvinylpyrrolidone (w/v), 1.25% sucrose (w/v), 0.05% PEG8000 (w/v), 0.2% BSA (w/v), and 0.05% Tween-20 (v/v)) and stored at 4 °C until use. The antibody–Au and antibody–Fe_3_O_4_ nanoparticle conjugates were obtained using the same procedure, but the antibody–Au nanoparticle conjugate solution was additionally centrifuged for 30 min at 12 000 × *g* and 4 °C.

### Pretreatment of the sample pad, conjugate pad, and nitrocellulose membrane

The sample pad, which was made from glass fiber (Shanghai Kinbio Tech Co., Ltd., Shanghai, China), was saturated with a buffer (pH 7.4) containing 20 mmol L^−1^ sodium borate, 1% (w/v) sucrose, 1% (w/v) BSA, 0.5% (v/v) Tween-20, and 0.05% (w/v) NaN_3_ in water for 40 min, dried at 37 °C and stored in its dried condition until use.

The conjugate pad (Shanghai Kinbio Tech Co., Ltd.), which was composed of polyester fiber, was immersed in a solution containing 2% (w/v) BSA, 3% (w/v) sucrose and 0.05% (w/v) NaN_3_ in water for 1 h and then dried at 37 °C. After 3 μL per strip of the antibody–Au, antibody–Au/Fe_3_O_4_ or antibody–Fe_3_O_4_ nanoparticle conjugate was placed onto the polyester fiber to be used as the conjugate pad, the conjugate pad was dried for 1 h at 37 °C and stored in its dried condition until use.

The nitrocellulose membrane (Shanghai Kinbio Tech Co., Ltd.) was treated with a buffer (pH 7.4) containing 10 mmol L^−1^ phosphate-buffered saline (PBS) solution, 3% (w/v) BSA, and 0.5% Tween-20 for 1 h and subjected to three 5 min washes with 0.1% Tween-20 in 10 mmol L^−1^ PBS. The membrane was then dried at 37 °C and stored in its dried condition until use.

### Assembly and analysis of the immunochromatography test strip

**Fig. 1:**
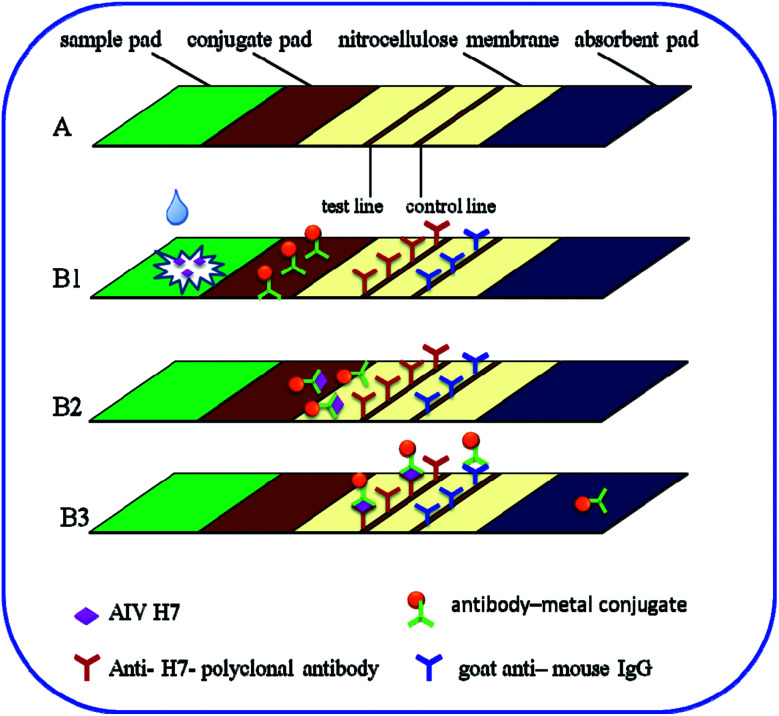
(A) Schematic illustration of the test strip and (B1–B3) the detection of AIV H7 using metal-based immunochromatography test strips. (B1) A sample containing AIV H7 is applied to the sample pad. (B2) AIV H7 combines with the antibody–metal conjugate and migrates along the nitrocellulose membrane by capillary action. (B3) The formed complexes continue to migrate along the membrane and are captured by the other type of anti-H7-polyclonal antibodies to form antibody–metal–AIV H7–anti-H7-polyclonal antibody complexes on the test line. As the liquid sample continues to migrate, the excess antibody–metal–AIV H7 complexes are captured by the secondary antibody (goat anti-mouse IgG), resulting in the accumulation of metal on the control line. The excess antibody–metal conjugates continue to migrate toward the absorption pad.

The immunochromatography test strip contains four main elements: a sample pad, a conjugate pad, a nitrocellulose membrane, and an absorbent pad. The strip was positioned in such a way that the ends of the elements overlapped, ensuring continuous flow of the developing solution from the sample pad to the absorbent pad *via* capillary action. As shown in Fig. 1A, the nitrocellulose membrane was pasted onto the center of the backing plate. The conjugate pad was also pasted onto the plate such that it overlapped the nitrocellulose membrane by 2 mm. The sample pad was pasted onto the same end such that its margin justified to the conjugate pad. The absorbent pad was then pasted onto the other end of the nitrocellulose membrane with the same 2 mm overlap. Afterward, 1 mg mL^−1^ anti-H7 polyclonal antibodies (Abcam) (*i.e.*, the test line) and a goat anti-mouse IgG antibody (Beijing Dingguo Biotechnology Co., Ltd., Beijing China) (*i.e.*, the control line) were immobilized onto the nitrocellulose membrane using an automatic dispenser (BioDot XYZ3000, USA). In particular, the anti-H7 polyclonal antibodies and the goat anti-mouse IgG antibody were dispensed onto the nitrocellulose membrane as the test line and the control line, respectively, with a width of 1 mm and a volume of 1 μL cm^−1^. The entire assembled plate was then cut into 3.5 mm strips and stored in its dried condition until use.

In total, 100 μL of sample solution containing the desired concentration of AIV H7 was added to the sample pad. Negative samples from specific-pathogen-free (SPF) chickens were used as controls. Both the sample and control solutions migrated toward the absorption pad *via* capillary action. After 15 min, the results could be assessed by eye: a negative result was indicated by a colorless test line, whereas a positive result presented as a brown/red test line. Both negative and positive results exhibited a red control line because the goat anti-mouse IgG is a nonspecific antibody that binds to all types of mouse antibodies.

## Results and discussion

### Principle of the method

In this study, the principle of the Au, Fe_3_O_4_ and Au/Fe_3_O_4_ core–shell nanoparticles is based on the sandwich immunoassay, as illustrated in Fig. 1. The immunochromatography test strip consists of a sample pad, a conjugate pad, a nitrocellulose membrane, and an absorption pad (Fig. 1A). All the components were assembled onto a plastic adhesive backing card. Sample solution containing the desired concentration of AIV H7 was added to the sample pad as illustrated in Fig. 1B1. The sample solution then migrated into the conjugation pad and bound to the antibody–metal nanoparticle conjugates based on the antibody–antigen interaction (Fig. 1B2). The formed complexes continued to migrate along the membrane and were captured by anti-H7 polyclonal antibodies immobilized beforehand on the nitrocellulose membrane to form antibody–metal–AIV H7–anti-H7 polyclonal antibody complexes, which resulted in the accumulation of metal nanoparticles on the test line. As the liquid sample continued to migrate, the excess antibody–metal–AIV H7–anti-H7 polyclonal antibody complexes were captured by the secondary antibody (goat anti-mouse IgG antibody), which resulted in the accumulation of metal nanoparticles on the control line. After 15 min, there were metal nanoparticles on the test line and the control line (Fig. 1B3). The results could be assessed by eye: a negative result was indicated by a colorless test line, whereas a positive result presented as a red/brown test line. Both negative and positive results exhibited a red/brown control line because the goat anti-mouse IgG is a nonspecific antibody that binds to all types of mouse antibodies. The excess antibody–metal conjugates continued to flow into the absorption pad at the end of the strip. If no metal nanoparticles accumulated on the control line, the test strip was invalidated and discarded. The fast immunoreaction and wash-free immunochromatography test strip detection made the assay rapid and easy to use.

### Optimization of the antibody–Au/Fe_3_O_4_ conjugates and the pretreatment solution

**Fig. 2:**
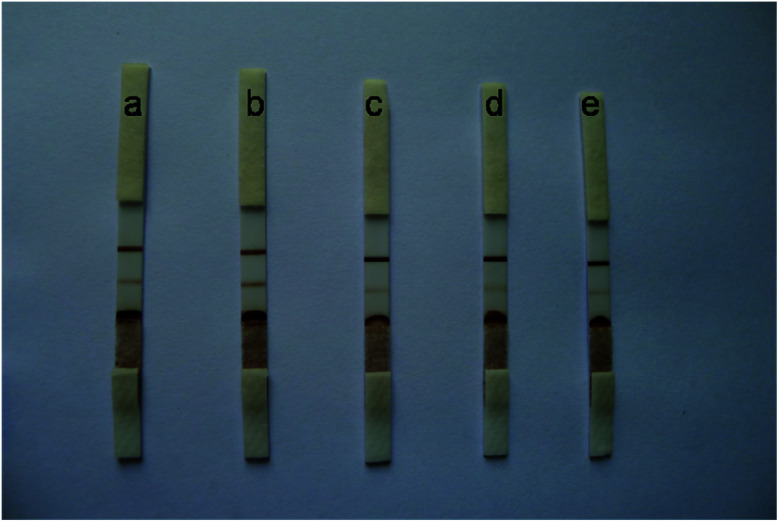
Different volumes of H7 monoclonal antibodies (1 mg mL^−1^) to 1 mL of Au/Fe_3_O_4_ nanoparticle solution: (a) 6 μL, (b) 5 μL, (c) 4 μL, (d) 3 μL, and (e) 2 μL.

The Au/Fe_3_O_4_ nanoparticle solution was adjusted to pH 8.5 with 0.1 mol L^−1^ K_2_CO_3_, and different volumes (*i.e.*, 6, 5, 4, 3, and 2 μL) of anti-H7 monoclonal antibodies (1 mg mL^−1^) were added to 1 mL of the Au/Fe_3_O_4_ nanoparticle solution to obtain different antibody–Au/Fe_3_O_4_ conjugates. These different antibody–Au/Fe_3_O_4_ conjugates were used for the construction of immunochromatography test strips for testing the same sample. The results are presented in Fig. 2. The color intensity detected using 5 μL of anti-H7 monoclonal antibodies yielded the best result.

**Fig. 3:**
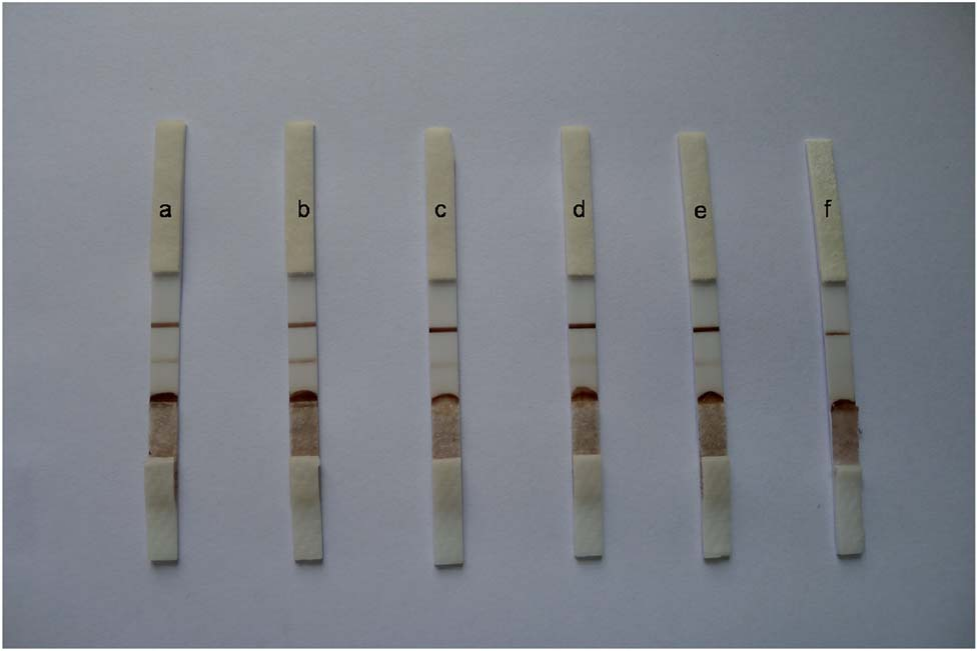
Optimization of the specificity of the immunochromatography test strip: (a) antibody–Au/Fe_3_O_4_ nanoparticle conjugates were stored in sodium phosphate buffer, (b) the sample pad, conjugate pad and nitrocellulose membrane were not pretreated, (c) the sample pad was not pretreated, (d) the conjugate pad was not pretreated, (e) the nitrocellulose membrane was not pretreated, and (f) the immunochromatography test strip developed in this study.

The specificity of the immunochromatography test strip might be influenced by many factors, such as storage of the antibody–Au/Fe_3_O_4_ nanoparticle conjugates and pretreatment of the sample pad, conjugate pad and nitrocellulose membrane. In our study, the antibody–Au/Fe_3_O_4_ nanoparticle conjugates were stored in storage buffer. The sample pad, conjugate pad and nitrocellulose membrane were pretreated according to the method described above. The effect of storage buffer was evaluated using sodium phosphate buffer instead of storage buffer to store the antibody–Au/Fe_3_O_4_ nanoparticle conjugates to detect negative samples from SPF chickens, which revealed nonspecific absorption, with two brown lines appearing on the nitrocellulose membrane (Fig. 3a). The sample pad, conjugate pad and nitrocellulose membrane without pretreatment were used to construct the immunochromatography test strip for detecting negative samples from SPF chickens, which revealed nonspecific absorption, with two brown lines appearing on the nitrocellulose membrane (Fig. 3b–e). In contrast, the immunochromatography test strip developed in this study generated a colorless test line due to its good specificity (Fig. 3f).

### Detection of AIV H7 using the immunochromatography test strip

**Fig. 4:**
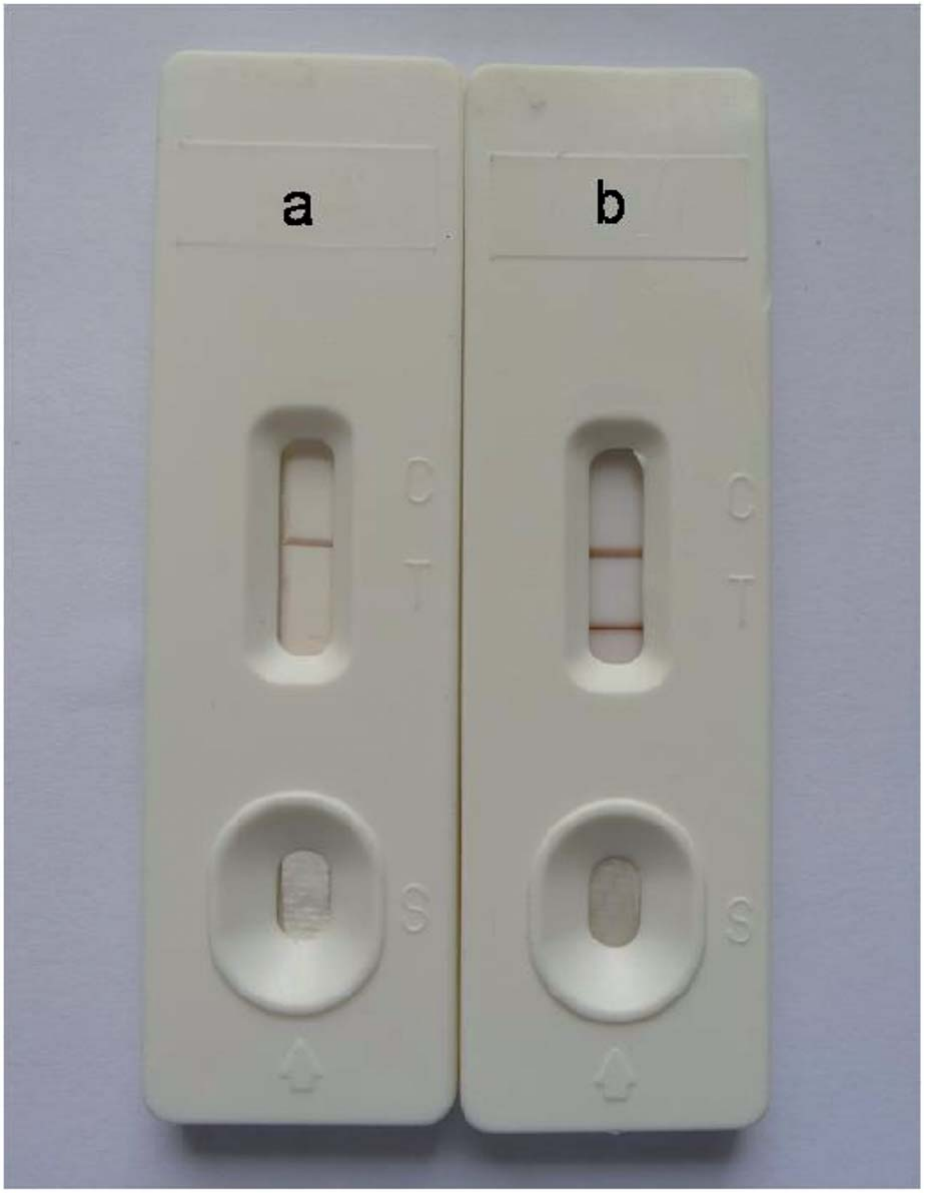
Results of the Au/Fe_3_O_4_ nanoparticle-based immunochromatography test strip for AIV H7. C: control line; T: test line. (a) A negative result (*i.e.*, samples from SPF chickens). (b) A positive result (*i.e.*, 10^5.5^ EID_50_ of AIV H7 (A/Duck/HK/47/76 (H7N2)).

**Fig. 5:**
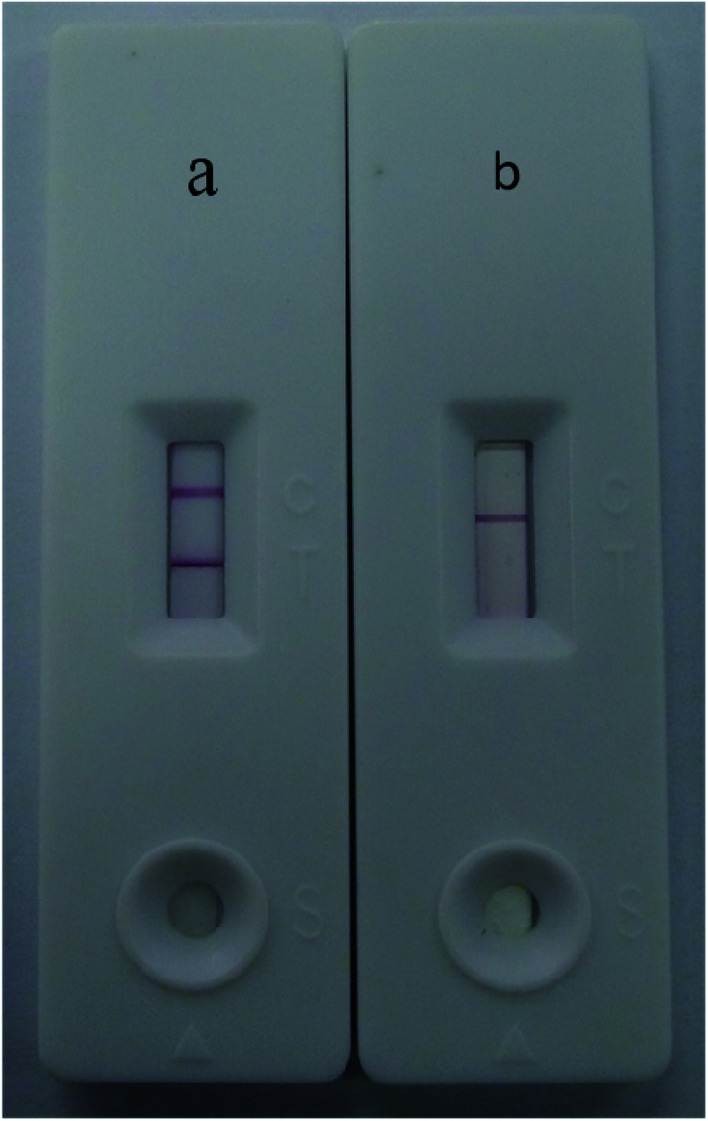
Results of the Au nanoparticle-based immunochromatography test strip for AIV H7. C: control line; T: test line. (a) A positive result (*i.e.*, 10^5.5^ EID_50_ of AIV H7 (A/Duck/HK/47/76, H7N2)) (b) A negative result (*i.e.*, samples from SPF chickens).

Under optimal conditions, the size of the test strip was 3.5 mm in width and 8 cm in length with a sample volume of 100 μL. The results of the Au/Fe_3_O_4_ core–shell nanoparticle-based immunochromatography strip test demonstrated that only one brown control line appeared for a negative test (*i.e.*, negative samples from SPF chickens). In contrast, AIV H7 tested positive and formed brown test and control lines on the nitrocellulose membrane (Fig. 4). The results of the Au nanoparticle-based immunochromatography strip test (Fig. 5) were similar to the Au/Fe_3_O_4_ core–shell nanoparticle-based immunochromatography strip test: only the color differed (red). However, the Fe_3_O_4_ nanoparticle-based immunochromatography strip test was considered invalid and was discarded because no Fe_3_O_4_ accumulated on the control line when negative samples were detected and no Fe_3_O_4_ accumulated on the control and test lines when positive samples were detected. The most likely reason for this result is that the Fe_3_O_4_ nanoparticles failed to label the anti-H7 monoclonal antibodies, whereas the anti-H7 monoclonal antibodies successfully immobilized onto the Au/Fe_3_O_4_ and Au nanoparticles because the Au/Fe_3_O_4_ and Au nanoparticles but not the Fe_3_O_4_ nanoparticles are highly compatible with antibodies. If Fe_3_O_4_ nanoparticles were selected as the labeling material for targeting antibodies to fabricate the immunochromatography test strip, they would need to be modified with effective functional groups (*e.g.*, a carbonyl group). Therefore, the Fe_3_O_4_ nanoparticle-based immunochromatography test strip would be more difficult to fabricate than the Au/Fe_3_O_4_ and Au nanoparticle-based immunochromatography test strips.

**Fig. 6:**
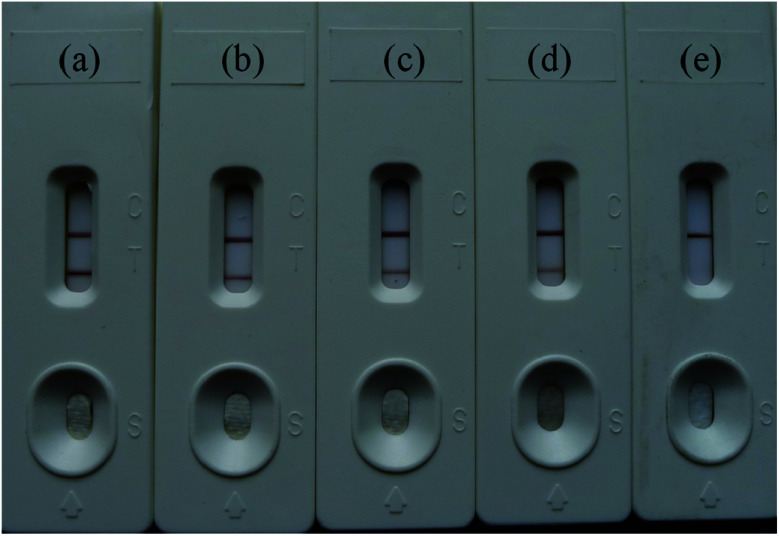
Detection of AIV H7 (A/Duck/HK/47/76 (H7N2)) using the Au/Fe_3_O_4_ nanoparticle-based immunochromatography strip test with AIV H7 titers of (a) 10^6.5^ EID_50_, (b) 10^5.5^ EID_50_, (c) 10^4.5^ EID_50_, (d) 10^3.5^ EID_50_, and (e) 10^2.5^ EID_50_.

**Fig. 7:**
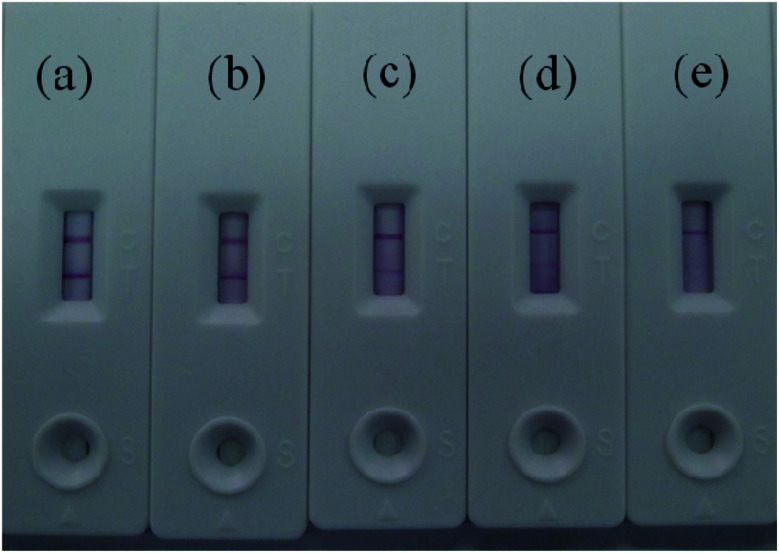
Detection of AIV H7 (A/Duck/HK/47/76 (H7N2)) using the Au nanoparticle-based immunochromatography strip test with AIV H7 titers of (a) 10^6.5^ EID_50_, (b) 10^5.5^ EID_50_, (c) 10^4.5^ EID_50_, (d) 10^3.5^ EID_50_, and (e) 10^2.5^ EID_50_.

The abilities of the Au/Fe_3_O_4_ and Au nanoparticle-based immunochromatography test strips to detect AIV H7 were evaluated at concentrations between 10^6.5^ EID_50_ and 10^2.5^ EID_50_. The experiments were repeated three times, and the results are presented in Fig. 6. Two brown lines appeared at concentrations of AIV H7as low as 10^3.5^ EID_50_, whereas only the control line appeared brown on the nitrocellulose membrane at concentrations less than 10^3.5^ EID_50_. Therefore, the limit of detection (LOD) of this method was 10^3.5^ EID_50_ for AIV H7 after evaluation by the naked eye within 15 min. When the antibody–Au nanoparticles conjugate was used as a label, the detection limit for AIV H7 was 10^4.5^ EID_50_ (Fig. 7). Compared with the antibody–Au nanoparticle conjugate, the use of the antibody–Au/Fe_3_O_4_ conjugate as the label is more sensitive. A comparison study between the Au/Fe_3_O_4_ immunochromatography test strip and other methods for AIV H7 detection is summarized in Table 1. It the results indicated that the developed Au/Fe_3_O_4_ immunochromatography test strip has acceptable sensitivity, with an advantage in rapid detection, intuitive, user-friendly and inexpensive and can truly achieve point-of-care testing. Our on-going research will focus on the application of the Au/Fe_3_O_4_ immunochromatography test strip for detection of other disease in the field.

**Table 1:** A comparison study between the Au/Fe_3_O_4_ immunochromatography test strip and other methods for AIV H7 detection^a^

Methods	Detection time	Detection limit	Advantages	Disadvantages	Reference
Virus isolation and identification	5–7 days	1 EID_50_ mL^−1^	“Gold standard”, sensitive, accurate	Labor-intensive and time consuming procedure;	5
RT-PCR	5 h	100 pg	Good in sensitive	Require expensive equipment, appropriate laboratory facilities and a trained technician;	6
Real-time RT-PCR	3 h	3.2 × 10^−4^ HAUs	Good in specificity and sensitivity	Expensive and complicated operation	7
ELISAs	3 h	10^3^ TCID_50_	Good in specificity	Many experimental steps including incubation and washing steps	8
RT-LAMP	1.5 h	42.47 copies/reaction	Simple instruments, good in sensitivity	High rate of false positives	9
Au/Fe_3_O_4_ immunochromatography test strip	15 min	10^3.5^ EID_50_	Rapid, low-cost, intuitive, user-friendly and truly realize point-of-care testing	Other disease detection will be needed for the on-going research	This study

a HAUs = Hemagglutination units; TCID_50_ = 50% Tissue Culture Infective Dose.

We also evaluated the shelf life of the Au/Fe_3_O_4_ nanoparticle-based immunochromatography test strip for field use. All Au/Fe_3_O_4_ nanoparticle-based immunochromatography test strips were stored at 4 °C for 90 days to test the sample concentration of 10^4.5^ EID_50_ and 10^3.5^ EID_50_ AIV H7. Negative samples from SPF chickens were used as controls. The results demonstrated that the Au/Fe_3_O_4_ nanoparticle-based immunochromatography test strip can be stored at 4 °C for 90 days and still continue to detect AIV H7 effectively.

To evaluate the specificity of the Au/Fe_3_O_4_ nanoparticle-based immunochromatography test strip, certain non-target samples, such as inactivated H7N9, H7N2, H7N7, H1N1, H2N3, H3N2, H4N6, H6N8, H8N4, H9N2, H10N3, H11N9, H12N5, H13N5, H14N5, H15N9, H16N3, infectious laryngotracheitis virus (ILTV), infectious bronchitis virus (IBV) and Newcastle disease virus (NDV), were tested. The experimental procedure was the same as that used for the H7 target. The experiments were repeated three times. The results are shown in Table 2. All the results of the AIV H7 sample tests were positive, and all results of the non-target sample tests were negative. These results demonstrate that the test strip is specific for AIV H7.

**Table 2:** Specificity of the Au/Fe_3_O_4_ nanoparticle-based immunochromatography test strip^a^

Avian pathogen	Source	Concentration		Results
		Virus titer (EID_50_)	HA titer	
A/chicken/BD135/2013 (H7N9)	CAU	10^5.5^		+
A/chicken PA/3979/97 (H7N2)	PU	10^4^		+
A/Chicken/NY/273874/03 (H7N2)	UCONN	10^4.5^		+
A/Duck/HK/47/76 (H7N2)	UHK	10^5.5^		+
A/Duck/42 846/07 (H7N7)	PU	10^4^		+
A/Duck/Guangxi/030D/2009 (H1N1)	GVRI		128	—
A/Duck/HK/77/76 d77/3 (H2N3)	UHK		64	—
A/Duck/Guangxi/M20/2009 (H3N2)	GVRI		64	—
A/Duck/Guangxi/070D/2010 (H4N6)	GVRI		32	—
A/Chicken/QT35/98 (H5N9)	PU		128	—
A/Duck/Guangxi/GXd-6/2010 (H6N8)	GVRI		64	—
A/Turkey/Ontario/6118/68 (H8N4)	UHK		128	—
A/Chicken/Guangxi/DX/2008 (H9N2)	GVRI		256	—
A/Duck/HK/876/80 (H10N3)	UHK		64	—
A/Duck/PA/2099/12 (H11N9)	PU		64	—
A/Duck/HK/862/80 (H12N5)	UHK		128	—
A/Gull/Md/704/77 (H13N5)	UHK		64	—
A/Mallard/Astrakhan/263/82 (H14N5)	UCONN		64	—
A/Shearwater/Western Australia/2576/79 (H15N9)	UCONN		64	—
A/Shorebird/Delaware/168/06 (H16N3)	CIVDC		64	—
ILTV (Benjing)	CIVDC	10^6^		—
IBV (Mass41)	CIVDC	10^5.2^		—
NDV (F48E9)	CIVDC		128	—

a PU = Pennsylvania State University, USA; CAU = China Agricultural University; UHK = University of Hong Kong, China; GVRI = Guangxi Veterinary Research Institute; CIVDC = China Institute of Veterinary Drug Control; UCONN = University of Connecticut, USA.

### Application of the Au/Fe_3_O_4_ nanoparticle-based immunochromatography test strip for the detection of AIV H7

The clinical samples were prepared in a viral transport medium composed of 0.05 M PBS containing penicillin (10 000 units per mL), streptomycin (10 mg mL^−1^), gentamycin (10 mg mL^−1^), kanamycin (10 mg mL^−1^) and 5% (v/v) fetal bovine serum and were placed in an ice box.

A total of 200 clinical swab samples were collected from chickens with permission from the owners of the live bird markets, and the samples were assayed using the optimized Au/Fe_3_O_4_ nanoparticle-based immunochromatography test strip: seven AIV H7-positive samples were detected and confirmed by virus isolation (virus isolations were prepared by inoculating SPF embryonated chicken eggs and were tested using a hemagglutination assay (HA) and a hemagglutination inhibition (HI) assay as described previously^25^), with the positive results being 100% comparable to those for virus isolation. Our immunochromatography test strip uses an Au/Fe_3_O_4_ core–shell nanoparticle whose Au nanoparticle shell makes it perfectly biocompatible and whose magnetic nanoparticle Fe_3_O_4_ core ensures it can be rapidly separated by a magnet. In contrast, magnetic-based immunochromatography strips require modification of the magnetic particle surface before labeling.^18^

## Conclusion

A novel approach for the rapid detection of AIV H7 using an immunochromatography test strip was successfully developed. We used Au/Fe_3_O_4_ core–shell nanoparticles as the label; these particles are easily and rapidly separated using a magnet during the labeling process, and the Au/Fe_3_O_4_ surface requires no modification prior to labeling. This assay, which had an LOD of 10^3.5^ EID_50_, provided a 10-fold lower LOD compared with an assay that used an antibody–Au conjugate label (LOD: 10^4.5^ EID_50_). The assay specifically detected AIV H7N2 and recombinant HA proteins of H7 subtypes, including H7N7 and H7N9, but did not react with non-H7 subtypes, including H1N1, H2N3, H3N2, H4N6, H6N8, H8N4, H9N2, H10N3, H11N9, H12N5, H13N5, H14N5, H15N9, H16N3, ILTV, IBV and NDV. Our assay was also successfully applied for the detection of AIV H7 in clinical samples through a single step and within 15 min. The results also demonstrated that the test strip can be stored at 4 °C for 90 days and continue to detect AIV H7 effectively. Therefore, the Au/Fe_3_O_4_ immunochromatography strip test reported herein, which is a rapid, simple and low-cost method, is a potentially valuable means for the detection and rapid clinical diagnosis of AIV H7. Consequently, it will be a very useful screening assay for the surveillance of AIV H7 in underequipped laboratories.

## Authors contributions

Zhixun Xie designed and coordinated the study. Jiaoling Huang designed the immunochromatography strip test, optimized the conditions of the immunochromatography strip test and finalized the analysis. All the other authors offered much help in the supplement of different clinical samples, shared previous experimental data and approved the final version of the manuscript.

## Conflicts of interest

There are no conflicts of interest to declare.

## Supplementary Material
